# Nanotechnology-Based Strategy to Upgrade the Performances of Plastic Flexible Film Waste

**DOI:** 10.3390/polym11050830

**Published:** 2019-05-08

**Authors:** Emilia Garofalo, Luciano Di Maio, Paola Scarfato, Fabrizio Di Gregorio, Loredana Incarnato

**Affiliations:** 1 Department of Industrial Engineering, University of Salerno, 84084 Fisciano, Italy; ldimaio@unisa.it (L.D.M.); pscarfato@unisa.it (P.S.); lincarnato@unisa.it (L.I.); 2 Conai–Italian National Consortium for Packaging Recycling, 20122 Milano, Italy; fabrizio.1981@hotmail.it

**Keywords:** flexible packaging, mechanical recycling, polymer blends, nanocomposites

## Abstract

The aim of this work was to improve the performances of Fil-s (film-small), a recycled material obtained from plastic flexible film waste that is made of polyethylene and a minor amount of polypropylene, with traces of polar contaminants (polyamides, maleic anhydride, etc.). The idea was to upgrade the material’s mechanical properties by applying a nanotechnology-based strategy that takes advantage of the composition of Fil-s. In particular, different amounts of copolyamide (CoPA) and its masterbatch with an organic-modified nanosilicate were melt compounded with Fil-s in a twin-screw extruder. The good affinity between Fil-s and CoPA, proved by means of spectroscopic and rheological analysis, allowed for the obtaining of a well-refined morphology for the neat and hybrid blends. This resulted in very interesting increments of the strain at break, which was particularly impressive (10 times higher) in the case of the blend with the lower amount of copolyamide masterbatch, but without sacrificing the stiffness and strength of Fil-s.

## 1. Introduction

Cheap, flexible, and multipurpose plastic has become the ubiquitous material of today’s fast-moving economy, but it poses substantial environmental problems due to its accumulation in ecosystems when disposed of improperly [1,2]. Ever-increasing attention on these negative aspects has stimulated initiatives to tackle these problems [3,4,5,6], especially with respect to packaging, as this represents the main application of plastics and makes up the largest share in the post-consumer plastic waste stream [7,8].

On the waste management side, the European Union has imposed a recycling target, which currently demands 22.5% of waste plastic packaging to be recycled [9], and that figure is proposed to increase to 55% by 2030 [10]. Nevertheless, Italy is one of the few countries in Europe that manages the recycling/recovery of all plastic packages, including flexible packaging, while the majority of other countries collect only those plastics that are easier to recycle, such as polyethylene terephthalate (PET) and high density polyethylene (HDPE) bottles.

Flexible packaging is fast and constantly expanding the range of its market applications due to specific features, such as (1) tailored barrier/protection; (2) customized shape, size, and format; and (3) light weight and light volume. Thus, it appears clear that the collecting, sorting, and recycling of post-consumer flexible packages represent necessary steps to hit the more stringent recycling target imposed by the European Union for 2030.

At present, recycling plastic flexible packages presents a number of challenges with low profits [11]. One major problem for producing recycled resins from this waste stream is the presence of different polymer types, which are not easy to separate, due to the similarity of their physical properties. They are often incompatible with each other and require different processing conditions. Moreover, several types of inorganic and/or organic contaminants may also be present. Consequently, the performances of the products, obtained from recycled flexible packaging, are not good.

In this study, we are concerned with the upgrading of Fil-s (film-small), which represents a fraction of the mixed plastic waste stream, obtained via the sorting and mechanical recycling of films of small size (<A3 format).

In our previous works [12,13,14], quality assessment of different batches of Fil-s was conducted, evidencing that this recycled material is mainly composed of polyethylene (LLDPE and LDPE) and a minor amount of polypropylene. Moreover, traces of other polymers, such as polyesters and polyamides, and of low-molecular-weight contaminants were also detected. A first strategy that we pursued to improve the mechanical properties of Fil-s concerned the addition of different types of nanofillers to the recycled material [12,13].

Solid nanoparticles typically have a relatively low cost and can offer the combination of improved properties and processing [15,16,17,18,19,20,21]. Compared with conventional micrometer-size particles, nanofillers not only add stiffness to polymers, but also introduce new energy-dissipation mechanisms [22,23], leading to an enhanced stiffness-to-toughness ratio. Moreover, in blend systems, they can act as compatibilizers [24,25,26,27] and morphology directors [27,28,29,30,31,32,33,34], depending on their distribution among the polymeric phases. In particular, the use of nanoparticles in post-consumer mixed polymeric materials was recently explored [35] and showed interesting potential as a waste management strategy, because it offers the combination of improved properties, ease of processing, and low cost. However, what appears clear from the recent literature on hybrid mixtures [26,27,28,29,30,31,32,33,34,35] and from our previous research activity on Fil-s [12,13], is that the great potentialities of nanoparticles can be only be exploited if a good affinity between the nanofiller and at least one component of the polymer blend exists.

In the present work, the strategy pursued to upgrade Fil-s was to first prepare a well-dispersed nanocomposite system and then to add it to the recycled material. In particular, a virgin copolyamide (CoPA) and an organo-modified sepiolite (PM15) were selected, because their good affinity was already proven in our previous research [36]. A masterbatch CoPA+20 wt % PM15 was produced by melt compounding using a twin-screw extruder. Subsequently, very low amounts (2.5 and 5 wt %) of both the neat CoPA and the nanocomposite masterbatch were mixed with Fil-s in the melt state. A good distribution/dispersion of the copolyamide phase in the recycled material was expected due to the presence of polar contaminants (traces of polyamide, maleic anhydride, and so on) inside Fil-s. The possible chemical interactions between Fil-s, CoPA, and the nanofiller were investigated by means of FTIR spectroscopic analysis, and their effect on the obtained morphologies for the neat (Fil-s/CoPA) and nanocomposite (Fil-s/CoPA/PM15) blends was also analyzed, together with their thermal and rheological properties. Finally, all the blends were extruded as ribbons, by means of a single-screw extruder, and submitted to mechanical characterization in tensile mode.

## 2. Materials and Methods

### 2.1. Materials

COREPLA (the Italian Consortium for the Collection and Recycling of Plastic Packages) supplied Fil-s. As extensively reported in our recent work [13], the physical, chemical, and rheological characterization of Fil-s evidenced that it is primarily constituted of polyethylene (LLDPE and LDPE) and a lesser fraction of polypropylene (about 12 wt %), with traces of other polymers, such as polyesters and polyamides, and of low-molecular-weight contaminants. In particular, a content of polar components inside Fil-s, equal to about 0.1 wt %, was determined by means of a non-aqueous back-titration procedure [13].

### 2.2. Production of Copolyamide-Based Nanocomposite Masterbatch

The nanocomposite masterbatch was prepared using a copolyamide 6/66 (supplied by Radici Group SpA, Bergamo, Italy, and denoted in the following as CoPA) as the polymer matrix and a needle-like silicate, the sepiolite Pansil PM15 (supplied by Tolsa Group, Madrid, Spain), which was organically modified by benzyl dimethyl hydrogenated tallow quaternary ammonium, as the nanofiller.

A masterbatch at 20 wt % of nanosilicate content was produced by melt compounding using a twin-screw extruder (Dr. Collin GmbH—model ZK 25-48D, Ebersberg, Germany) with co-rotating intermeshing screws (D_screw_ = 25 mm, L/D = 42). A screw speed of 220 rpm and a temperature profile of 100–245–245–245–245–230–230–230 °C (from hopper to die) were used. The ribbons of the neat matrix and the masterbatch were produced by means of a Brabender DCE 330 (GmbH & Co KG, Duisburg, Germany) single-screw extruder (D_screw_ = 20 mm and L/D = 20) using a screw speed of 10 rpm and a temperature profile of 230–245–215 °C (from hopper to die). Prior to processing, the materials were dried in a vacuum oven at 110 °C for 18 h to avoid bubble formation and polymer degradation during processing.

### 2.3. Production of Neat and Nanocomposite Fil-s/Copolyamide Blends

Different amounts (2.5 and 5 wt %) of both the neat copolyamide and its nanocomposite at 20% of PM15 were added to Fil-s in the same twin-screw extruder, having previously dried the materials at 110 °C and 70 °C for 18 h, respectively. The temperature profile of the extruder was set at 150–240–240–240–240–240–240–210 °C (from hopper to die), and a screw speed of 100 rpm was imposed.

Then, the obtained Fil-s/CoPA blends were extruded in the form of ribbons by means of the same Brabender single-screw extruder. A screw speed of 20 rpm and a temperature profile of 200–200–230 °C (from hopper to die) were selected.

The effective level of silicate in each extruded nanocomposite system was determined by drying the samples at 100 °C for 18 h under vacuum and weighing them before placing them in a furnace at 900 °C for 45 min in air. The amount of residue was corrected for the loss of organic component present in sepiolite PM15. Each determination was repeated on five specimens to obtain statistical silicate loading values, which are reported in Table 1.

### 2.4. Characterization Techniques

Fourier-transform infrared spectroscopy (FTIR) measurements were carried out in the range of 4000–650 cm^−1^ with a Nexus ThermoNicolet spectrometer (Thermo Fischer Scientific, USA), using a SmartPerformer accessory for (Attenuated Total Reflection) ATR analyses. The spectra were collected at 2 cm^−1^ spectral resolution, and 64 scans were co-added.

Differential scanning calorimetry (DSC) analyses were performed using a DSC30 Mettler calorimeter (Mettler-Toledo International Inc., Novate Milanese MI, Italy) according to the following thermal cycle: a first heating at 10 °C/min from 0 to 250 °C; an isotherm at 250 °C for 5 min; a cooling to 0 °C; and a re-heating to 250 °C at the same scan rate. DSC measurements were carried out in a nitrogen atmosphere, using aluminum crucibles filled with about 10 mg of the samples.

Scanning electron microscopy (SEM) analysis was conducted using a Zeiss EVO MA10 microscope with a secondary electron detector (Carl Zeiss SMT AG, München-Hallbergmoos, Germany), operating at 14 kV. The images were taken on specimens cryo-fractured in liquid nitrogen and sputter-coated with a 200–440 Å thick gold layer by means of a Leica EMSCD005 metallizator.

Measurements of CoPA droplets diameter were performed on about 1000 particles, using a free Image Editor software (GIMP). Number (*d_n_*) and volume (*d_v_*) average diameters and polydispersity (D) were calculated using Equations (1)–(3).

(1) $$d_{n}=\frac{\sum n_{i}d_{i}}{\sum n_{i}}$$

(2) $$d_{v}=\frac{\sum n_{i}d_{i}^{4}}{\sum n_{i}d_{i}^{3}}$$

(3) $$D=\frac{d_{v}}{d_{n}}$$

where *n_i_* is the number of droplets of diameter *d_i_*.

Rheological experiments in oscillatory mode were conducted with a rotational rheometer ARES (Rheometric Scientific, USA) under a nitrogen atmosphere. The tests were performed at 250 °C in an angular frequency range from 0.1 to 100 rad/s, using 25 mm diameter parallel plates. A strain amplitude of 5% was proven to ensure linear viscoelasticity during the dynamic rheological measurements.

Tensile mechanical tests were performed, according to ASTM D882, by means of a CMT4000 Series dynamometer (SANS, Shenzhen, China). The specimens were submitted to a crosshead speed of 5 mm/min to measure the Young’s modulus and 500 mm/min to determine the mechanical properties at break. The data were mediated on 10 samples for each type of system analyzed.

## 3. Results and Discussion

In the following, the unfilled Fil-s/CoPA blends were first characterized by spectroscopic analysis, in order to highlight the potential interactions between the recycled material and the copolyamide phase that, in turn, affect the distribution/dispersion of CoPA particles inside Fil-s. The resulting rheological and thermal properties were also studied.

In the next paragraph, the nanocomposite Fil-s/CoPA/PM15 blends were analyzed. In particular, the fundamental phenomena that govern the morphology of hybrid mixtures (made of more than one polymeric phase and an inorganic nanofiller), such as thermodynamics and/or kinetic effects, as well as the nanoparticles localization, were investigated.

All the neat and nanocomposite blends were finally extruded as ribbons, whose tensile mechanical properties were measured and compared in the last paragraph of this section.

### 3.1. Neat Fil-s/CoPA Blends

The FTIR band assignments for Fil-s and CoPA are listed in Table 2 and Table 3, respectively [12,13,37,38].

**Figure 1:**
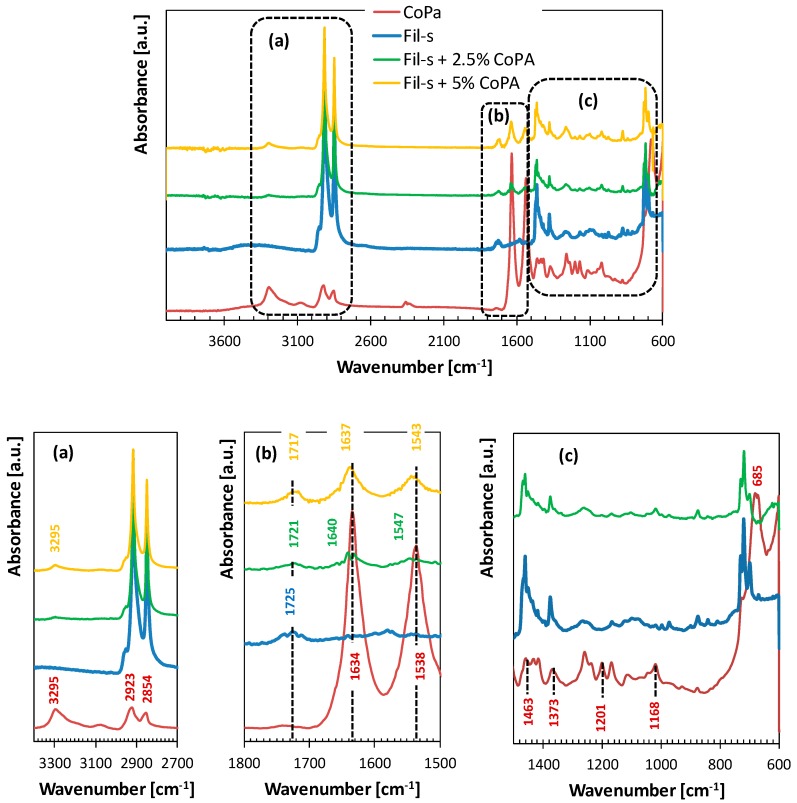
Comparison of FTIR/ATR spectra of Fil-s, the neat copolyamide, and their blends.

The spectrum of Fil-s is compared in Figure 1 with the spectra of the neat copolyamide and Fil-s/CoPA blends.

In the FTIR spectra of both the Fil-s/CoPA blends, the main absorption peaks of Fil-s remain essentially unaltered, and some typical vibration bands of CoPA can be clearly observed. In particular, the two strong absorption bands, at 1634 and 1538 cm^−1^, attributed to the C=O stretching vibration and the N–H bending vibration, respectively, are shifted toward higher wavenumbers in the blends compared with the neat copolyamide (Figure 1b). In the case of Fil-s + 2.5%CoPA blend, the CO band is split into two signals, one of which remains at the original vibration wavenumber. The other is shifted about 6 cm^−1^ higher, while the NH band is simply shifted of about 9 cm^−1^ higher than the neat CoPA. Less pronounced shifts of the same FTIR signals can be observed for the blend Fil-s + 5%CoPA (Figure 1b). The shift of these bands is indicative of the interactions between the polar contaminants inside Fil-s and the copolyamide.

**Figure 2:**
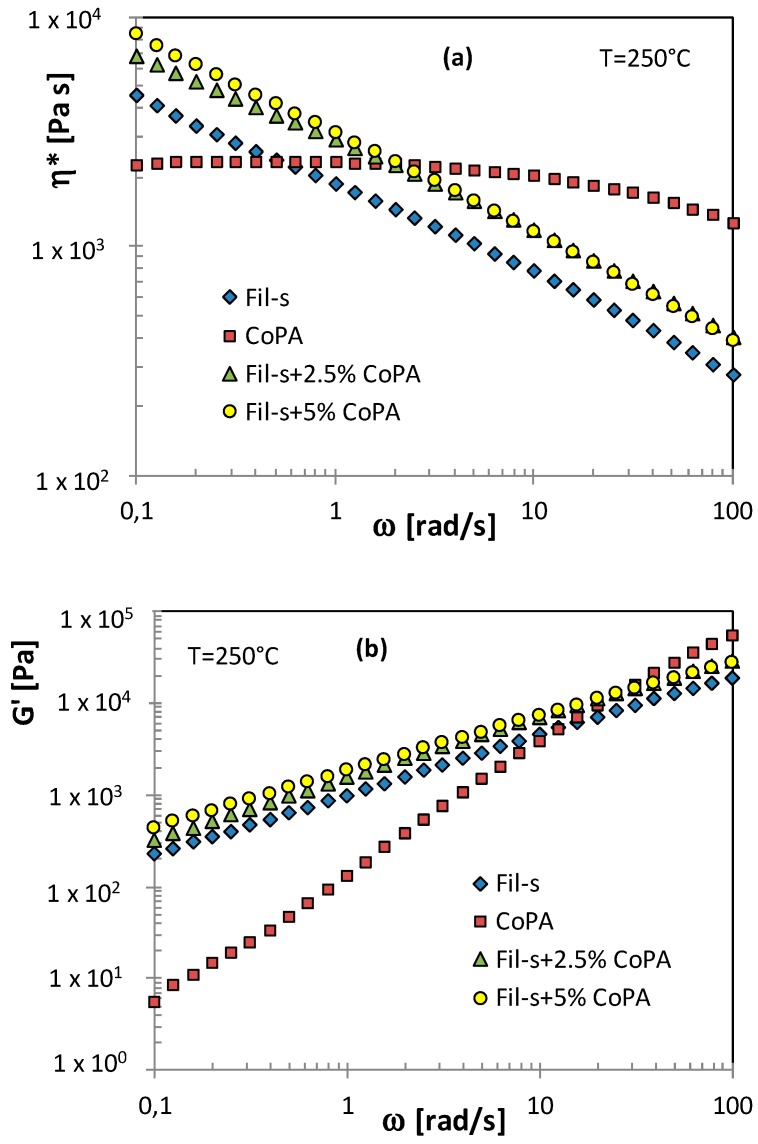

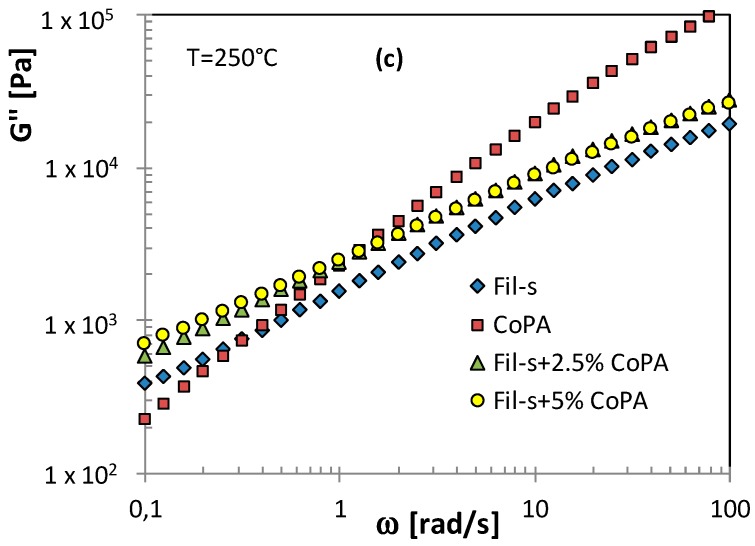
Comparison of the dynamic viscoelastic properties of Fil-s, the neat copolyamide, and their blends: (**a**) complex viscosity; (**b**) storage modulus; and (**c**) loss modulus.

With the aim to investigate the flow behavior of the analyzed systems, rheological tests were carried out. Figure 2 reports the results of frequency sweep experiments in the linear viscoelastic regime on both the neat components (Fil-s and CoPA) and their blends.

Fil-s shows a very pronounced shear thinning behavior, already at low frequencies, while for the neat copolyamide, a wide Newtonian plateau can be observed and a slight decrease of η* appears only at the higher frequencies analyzed. The Fil-s/CoPA blends exhibit viscosity plots similar to that of the recycled material but at significantly higher values of η*. In particular, it is worth noting that, in the low frequency range, the blends show higher complex viscosity values compared with both Fil-s and CoPA. This result can be attributed to the physical interactions across the interface between CoPA and the polar functional groups of Fil-s, as detected by FTIR. These interactions further increase the effect of interfacial elasticity [39,40] between the different phases of the blends.

Moreover, it can be observed that doubling the content of CoPA (5 wt %) inside the blend does not cause a further significant increment of η* compared with the system Fil-s + 2.5% CoPA. This latter outcome is in agreement with the FTIR data obtained for the blend Fil-s + 5% CoPA, which highlight the less pronounced interactions between the mixture components in the presence of a higher amount of the dispersed phase. This suggests that the functional polar groups inside Fil-s are probably saturated with the lower content of the copolyamide [39].

Looking at the storage and loss moduli plots (Figure 2b,c), both the neat copolyamide and Fil-s show a predominant viscous behavior in the entire frequency range, particularly in the case of CoPA. The Fil-s/CoPA blends display G’ and G’’ trends similar to the corresponding plots of Fil-s but at higher values, as already observed for the complex viscosity. Moreover, the characteristic shoulder of the storage modulus curve that, in a polymer blend, is representative of the droplet relaxation [39,40] does not clearly appear for the Fil-s/CoPA systems in the frequency range analyzed, probably due to the very low amounts of the copolyamide phase.

**Figure 3:**
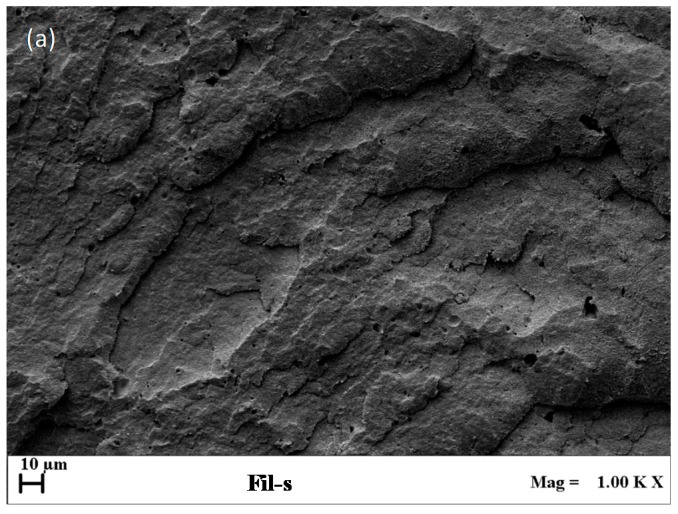

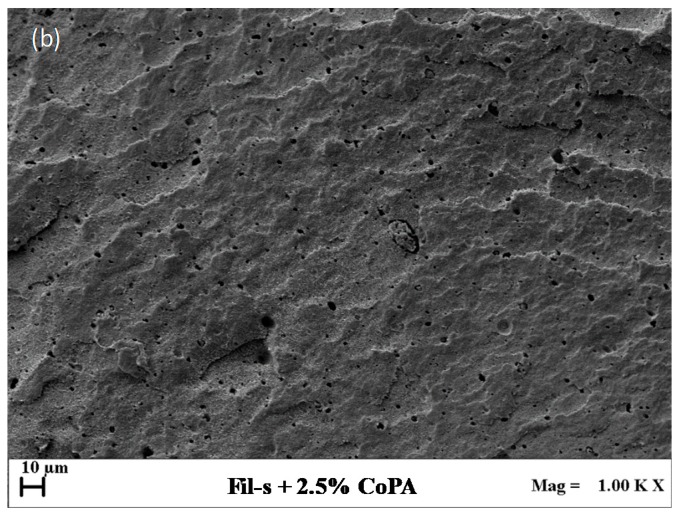
SEM images, captured on the sections of the samples cryo-fractured and etched with formic acid, for (**a**) neat Fil-s and (**b**) Fil-s + 2.5%CoPA blend.

The affinity between the recycled material and the virgin CoPA, in turn, affects the morphology of their blends. In Figure 3a,b, SEM images of Fil-s and Fil-s + 2.5%CoPA blend are reported.

The sections of these samples were cryo-fractured and etched with formic acid in order to dissolve the polyamide phase; thus, the holes, which can be observed in the images, represent the CoPA droplets dispersed inside Fil-s. In particular, some holes are clearly visible even in the neat Fil-s, confirming the presence in the recycled material of traces of polyamides. A well-refined morphology was obtained in the case of Fil-s+2.5% CoPA blend, as evidenced by the small CoPA particle sizes (*d_n_* = 0.89 μm and *d_v_* = 1.87 μm) and the quite narrow particle size distribution (D = 2.09).

**Figure 4:**
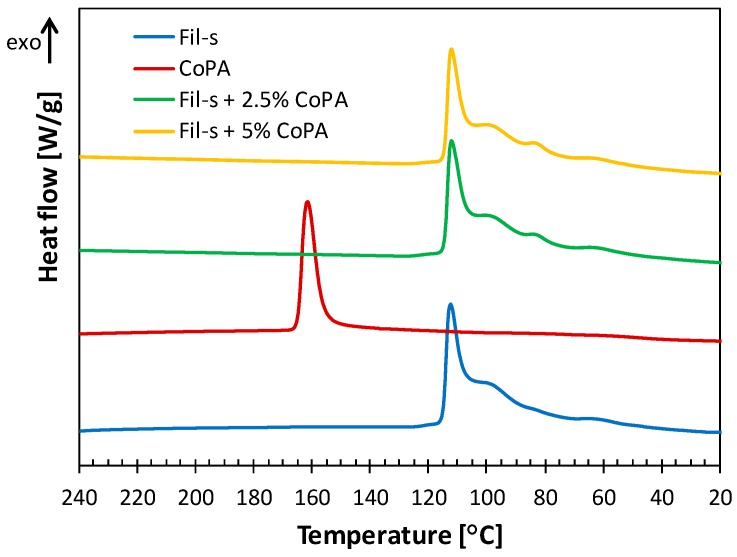
Comparison of differential scanning colorimetry (DSC) cooling thermograms of Fil-s, CoPA, and their blends.

In order to analyze the effect of the obtained CoPA dispersion on the crystallization behavior of the blends, DSC experiments were carried out. The cooling scans for the Fil-s/CoPA samples are compared with the corresponding ones of the neat components in Figure 4.

The copolyamide alone shows a well-defined crystallization peak at 162 °C. Instead, Fil-s has a more complex crystallization behavior with an intense peak at 112 °C and a quite pronounced shoulder at 99 °C. According to literature [41,42], this multiple peak can be reasonably associated with the crystallization of the polyethylene phase inside Fil-s, to which the crystallization of the PP phase is probably overlapped. This outcome can be explained in terms of the fractionated crystallization process [42]. Regarding the blends, their crystallization behavior is comparable to that of Fil-s alone. The absence of the exothermic crystallization CoPA peak may be due to both the low amounts of CoPA and its very fine dispersion inside Fil-s.

### 3.2. Nanocomposite Fil-s/CoPA/PM15 Blends

**Figure 5:**
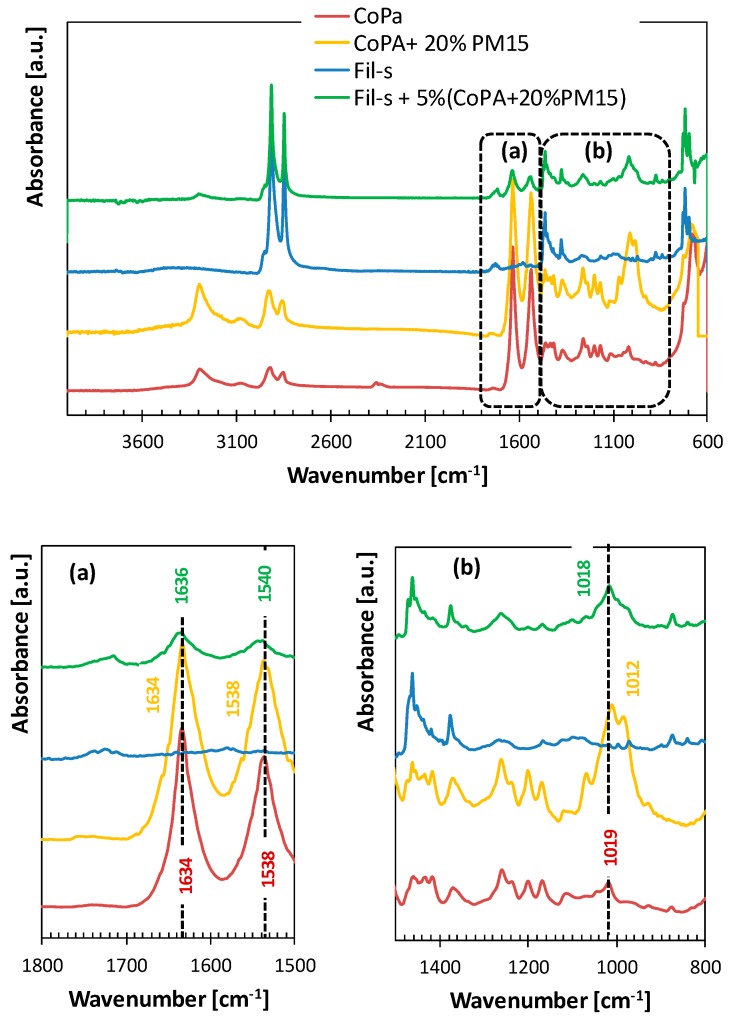
Comparison of FTIR/ATR spectra of Fil-s, neat copolyamide, copolyamide masterbatch, and the blend Fil-s + 5%(CoPA + 20%PM15).

Compared with the blend Fil-s + 5%CoPA (Figure 1), the FTIR spectrum of the sample Fil-s + 5%(CoPA + 20%PM15) (Figure 5) shows a quite pronounced absorption peak at about 1018 cm^−1^, which can be related to the Si–O–Si plane vibrations of the sepiolite [43].

Moreover, in the nanocomposite blend the characteristic C=O and N–H bands of the copolyamide evidence less pronounced shifts towards higher wavenumbers, 1636 and 1540 cm^−1^, respectively. This latter outcome can be attributed to reduced interactions between Fil-s and CoPA, reasonably due to the presence of some particles of sepiolite at the interface [38].

**Figure 6:**
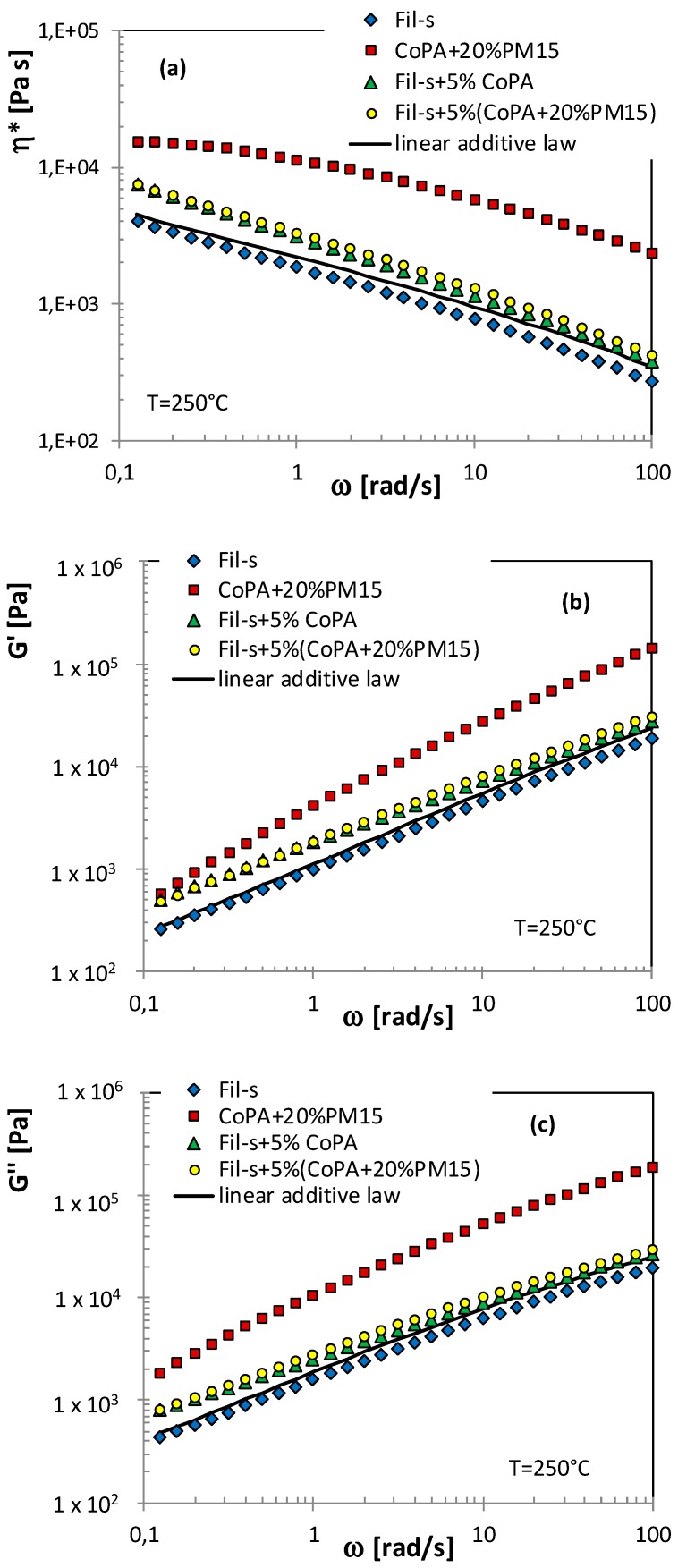
Comparison of the dynamic viscoelastic properties of Fil-s, the nanocomposite copolyamide masterbatch, and their blend; the solid lines represent the blend properties as predicted by a simple linear additive law: (**a**) complex viscosity; (**b**) storage modulus; and (**c**) loss modulus.

To evidence possible interactions between Fil-s and the copolyamide masterbatch and also to obtain information about the state of the nanosilicate dispersion inside the polymer phases, dynamic rheological tests were carried out on the blend Fil-s + 5%(CoPA + 20%PM15) and its components (Figure 6).

Comparing the trends of the viscoelastic properties for the neat Fil-s/CoPA and the nanocomposite Fil-s/CoPA/PM15 blends, no significant differences can be deduced due to the very low amounts of the nanosilicate (<1 wt %) inside the systems produced. Moreover, in the whole range of frequencies analyzed, the values of the dynamic properties η*, G’, and G’’ of the hybrid blend fall between the corresponding data of Fil-s and the CoPA masterbatch. However, as already evidenced for the unfilled Fil-s/CoPA systems, a synergistic effect among the blend components can still be deduced, because significantly higher dynamic rheological data can be observed for the blend Fil-s + 5%(CoPA + 20%PM15) compared with the ones predicted by a simple linear additive rule (Figure 6), particularly in the low-frequency range.

Regarding the selective localization of the nanoparticles inside the hybrid blends, the silicate surface chemistry and the polarity of the polymer phases will determine the affinity between components and therefore, the nanofillers migration. On the thermodynamic point of view, the wetting coefficient *ω_AB_* (Equation (4)) is generally used to deduce the nanoparticles location:

(4) $$\omega_{AB}=\frac{\gamma_{fB}-\gamma_{fA}}{\gamma_{AB}}$$

where *γ_fB_*, *γ_fA_*, and *γ_AB_* represent the interfacial tension between the filler and polymer B, the filler and polymer A, and the two polymers A and B, respectively.

As described by Sumita et al. [44] and afterwards by Fenouillot et al. [33], if *ω_AB_* >1, nanofillers preferentially locate in polymer A; if *ω_AB_* < −1, they are present only in polymer B; and when –1< *ω_AB_* <1, nanofillers are situated at the interface between the two polymers.

The interfacial energy can be calculated according to the harmonic mean (Equation (5)) or the geometric mean (Equation (6)) [45]:

(5) $$\gamma_{AB}=\gamma_{A}+\gamma_{B}-4\left(\frac{\gamma_{A}^{d}\gamma_{B}^{d}}{\gamma_{A}^{d}+\gamma_{B}^{d}}+\frac{\gamma_{A}^{p}\gamma_{B}^{p}}{\gamma_{A}^{p}+\gamma_{B}^{p}}\right)$$

(6) $$\gamma_{AB}=\gamma_{A}+\gamma_{B}-2\left(\sqrt{\gamma_{A}^{d}\gamma_{B}^{d}}+\sqrt{\gamma_{A}^{p}\gamma_{B}^{p}}\right).$$

In this work, surface energy values for CoPA and sepiolite PM15 (Table 4) were taken from the literature [46] and were used to calculate the interfacial tensions, reported in Table 5. In the case of Fil-s, an approximate value of the surface tension was also assumed from the literature, considering that this recycled material mainly consists of polyethylene.

Based on the calculated interfacial energies (Table 5), the wetting coefficient was determined according to Equation (6) and was equal to 0.30 and 0.39 using the harmonic mean and the geometric mean, respectively. Therefore, the theoretical calculation evidences that the needle-like nanofillers preferentially locate at the interface, as already indirectly suggested by the FTIR results (Figure 5).

However, the estimation of the wettability coefficient is not completely exhaustive to evaluate the final nanofiller localization among the polymer phases. In fact, in the molten state, the thermodynamic equilibrium may be difficult to attain due to the generally high viscosity of polymers. Moreover, shear induced dispersion and collisions between nanoparticles and dispersed droplets during processing also have to be taken into account [26,27,28].

**Figure 7:**
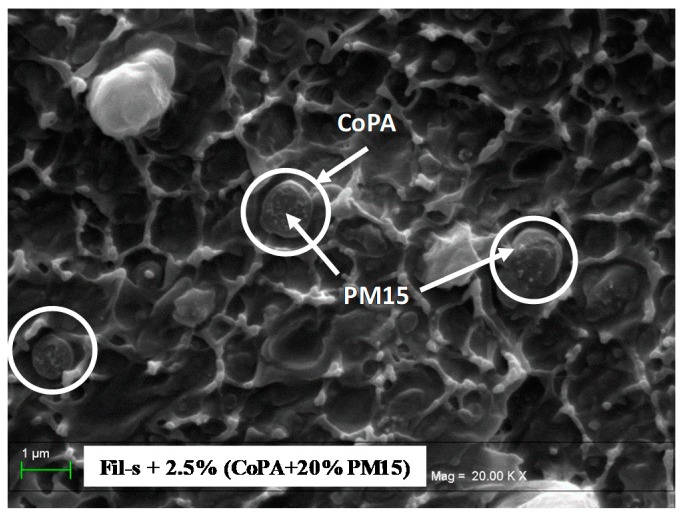
SEM image of the blend Fil-s + 2.5%(CoPA + 20%PM15). The copolyamide phase is evidenced inside the white circular lines.

In this particular case, first, the nanofillers were compounded with the copolyamide, and afterwards, the nanocomposite masterbatch was added to Fil-s. Furthermore, due to the high viscosity of the masterbatch CoPA + 20%PM15 (Figure 6), the migration of the inorganic particles from the copolyamide phase towards the interface with Fil-s is probably slow, and consequently, the equilibrium, dictated by the wetting parameter, may be partially reached even after reasonable mixing time. In this respect, the SEM micrograph of the blend Fil-s + 2.5%(CoPA + 20%PM15), reported in Figure 7, clearly shows some sepiolite particles inside the copolyamide phase (this latter being evidenced in the picture within the white circles).

In particular, the nanofillers appear as bright spots with circular shapes, representing the projection of the needle-like particles in the plane of the cross section.

**Figure 8:**
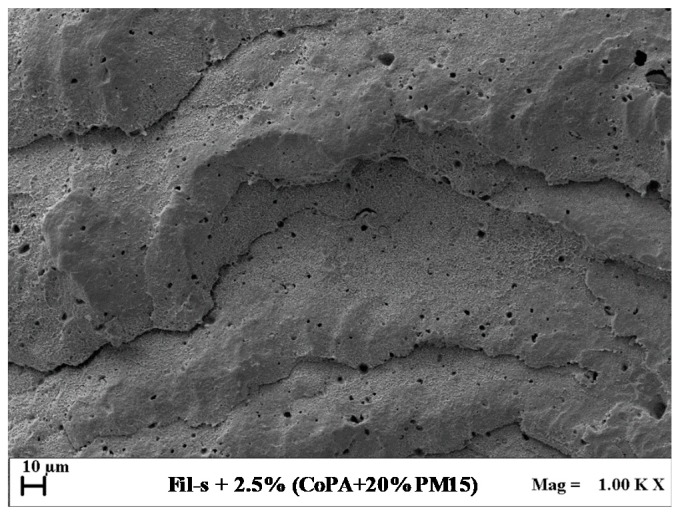
SEM image of the sample Fil-s + 2.5%(CoPA+20%PM15) captured on the section cryo-fractured and etched with formic acid.

The balance of the tripartite interactions between Fil-s, the copolyamide phase, and the organo-modified clay resulted, however, in a well-refined morphology of the blend Fil-s + 2.5%(CoPA + 20%PM15), as shown by the SEM micrograph of this latter sample, reported in Figure 8.

**Figure 9:**
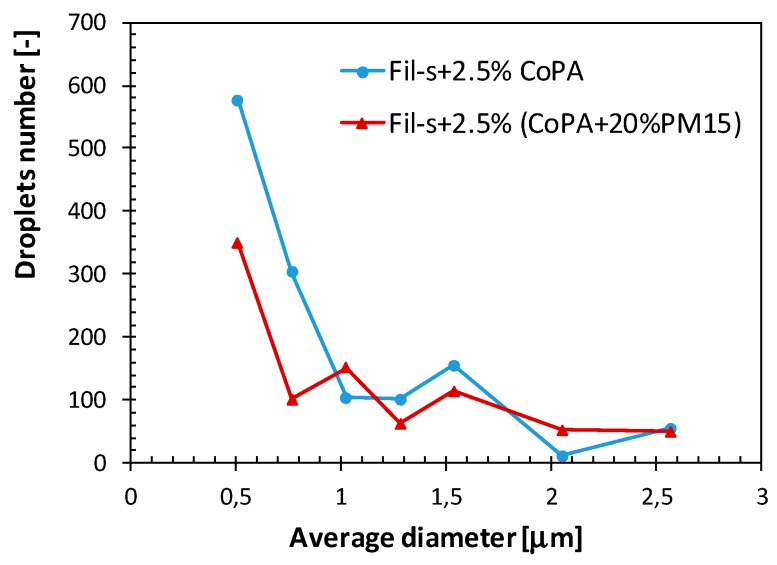
Particle size distribution of the CoPA dispersed phase for the unfilled and nanocomposite Fil-s/CoPA blends.

In particular, compared with the blend Fil-s + 2.5%CoPA, slightly higher values of the number average (*d_n_*) and volume average (*d_v_*) particles diameters (1.03 and 1.96 μm, respectively) were obtained for the nanocomposite system Fil-s + 2.5%(CoPA + 20%PM15). Moreover, this latter blend is characterized by a narrower particles size distribution (D = 1.91) and a significantly lower number of dispersed phase droplets with diameter less than 1 μm (Figure 9).

As already evidenced for the blends with the neat copolyamide, in the nanocomposite blends the fine CoPA dispersion does not affect the crystallization behavior of the multiphase systems, with no significant changes in the principal thermal parameters (crystallization temperatures and enthalpies) compared with the Fil-s alone (Table 6).

### 3.3. Mechanical Performances of Neat and Nanocomposite CoPA/Fil-s Blends

The blends of Fil-s with different amounts of unfilled and nanocomposite CoPA were extruded by means of a single-screw extruder in the form of ribbons, which were subsequently submitted to tensile tests. In Table 7, the principal mechanical properties of all the specimens analyzed are reported.

As extensively explained in our previous work [36], the presence of sepiolite determines a very pronounced increment of both the stiffness and tensile strength of the nanocomposite system CoPA + 20%PM15 compared with the neat copolyamide. Moreover, a significant reduction of the CoPA ductility due to the high sepiolite percentage can be observed. Another important mechanical property to take into account is the yield stress that can be strongly related to the polymer/clay affinity. To this regard, a significant enhancement in this mechanical parameter was shown by the masterbatch CoPA + 20%PM15. This result can be attributed to a good interaction between the matrix and the nanofiller due to the formation of hydrogen bonds between the silanol groups Si-OH of the sepiolite and the CO groups of the copolyamide.

The addition of the neat CoPA inside Fil-s determines a slight decrease of the Young’s modulus of the blend Fil-s + 2.5%CoPA relative to the recycled material but a very significant improvement in ductility (6 times higher). The blend Fil-s + 2.5%(CoPA + 20%PM15) shows an even more pronounced increment of the deformation at break (10 times higher) without sacrificing the stiffness and the strength of the neat Fil-s.

Doubling the amount (5 wt %) of CoPA in the blend, a remarkable increase in ductility of Fil-s (about 4 times higher) is still obtained, but it is less significant compared with the system Fil-s + 2.5% CoPA. For the hybrid blend Fil-s + 5%(CoPA + 20%PM15), a further reduction of the strain at break is observed. However, compared with the neat Fil-s, a 100% increase in ductility can be still evidenced, together with a concurrent enhancement in stiffness (about 18%), despite the very low amount of nanofiller (less than 1 wt %) in the blend (Table 1).

As in the case of rubber-toughened plastics [47], most likely, the CoPA droplets inside the blends act as craze initiators, controlling their growth and preventing the early failure of Fil-s alone due to large craze formation and breakdown [48]. It has been experimentally demonstrated in polymer matrices failing by crazing that the optimum rubber particle size is in the range of 1–5 μm [49,50]. In particular, Donald and Kramer [50] evidenced that crazes are rarely nucleated from particle sizes less than about 1 μm.

Thus, to obtain an efficient toughening mechanism a fine dispersion of the copolyamide phase throughout the recycled material is of fundamental importance. Moreover, an appropriate adhesion between the dispersed particles and the matrix is also necessary; in fact, a widespread damage of the dispersed phase fibrils due to break or debonding will lead to an immediate fracture of the material.

The Fil-s/CoPA blends meet both these requirements, as clearly evidenced by the spectroscopic (Figure 1) and morphological analysis (Figure 3) of the specimens. In particular, the more effective interaction between Fil-s and the neat copolyamide in the blend Fil-s + 2.5%CoPA accounts for the more significant ductility enhancement of the recycled material, compared with the blend Fil-s + 5%CoPA (Table 7).

The better ductile behavior of the hybrid blend Fil-s + 2.5%(CoPA + 20%PM15) relative to the system Fil-s + 2.5%CoPA, can be partly attributed to the narrower CoPA particles size distribution with a significantly lower number of sub-micron droplets (*d_n_* < 1 μm) (Figure 9). Moreover, the cavitation of the nanocomposite CoPA droplets probably leads to the formation of fibrils, which, stabilized by the rigid inclusions, favors the deformation of the matrix and slows down the breaking process, helping to support part of the stress.

## 4. Conclusions

The aim of this study was the upgrading of Fil-s, a mixed polyolefin recycled material obtained from post-consumer films of small size. In particular, different amounts of a virgin copolyamide and its nanocomposite masterbatch (CoPA + 20%PM15) were melt compounded with this recycled material, in order to combine the advantages of the addition of a high-performance plastic and the merits of polymer nanocomposites. The good affinity between CoPA and Fil-s was proved by both spectroscopic and rheological analysis, and this led to a well-refined morphology of Fil-s/CoPA systems, particularly in the case of the blend with the lower content (2.5% in weight) of CoPA. In fact, it was evidenced that this copolyamide amount was enough to saturate the interactions with the polar contaminants inside Fil-s, while a higher CoPA content tended to coalesce in the blend.

With regards to the hybrid mixture Fil-s + 2.5%(CoPA + 20%PM15), the balance of the tripartite interactions between the recycled matrix, the dispersed copolyamide phase, and the organo-modified clay also resulted in a fine blend morphology. In particular, this latter was characterized by a narrower particle size distribution and a significantly lower number of sub-micron CoPA droplets compared with the unfilled corresponding blend Fil-s + 2.5%CoPA. As a consequence, a more effective toughening mechanism (through craze formation) can be supposed for the nanocomposite blend Fil-s + 2.5%(CoPA + 20%PM15). This resulted in a very significant increase in ductility (10 times higher) compared with the neat Fil-s but without sacrificing the stiffness and the strength of the recycled material.

## Figures and Tables

**Table 1:** Actual copolyamide (CoPA) and nanofiller (PM15) contents in the blends with Fil-s.

SAMPLE	Actual CoPA Content [wt %]	Actual PM15 Content [wt %]
CoPA+20%PM15	–	18.03 ± 0.08
Fil-s + 2.5% (CoPA+20% PM15)	1.67 ± 0.21	0.30 ± 0.04
Fil-s + 5% (CoPA+20% PM15)	5.28 ± 0.23	0.95 ± 0.04

**Table 2:** FTIR frequencies and vibrational assignments for Fil-s.

Wavenumber (cm^−1^)	Assignment
3500–3200	–OH and –NH stretching
2915	–CH_2_ asymmetric stretching
2847	–CH_2_ symmetric stretching
16001–565	conjugated (C=C) stretching, –NH stretching
1462	–CH_2_ scissoring
1376	–CH_3_ scissoring
740–690	–CH_2_ rocking, (=C–H) bending, –NH stretching

**Table 3:** FTIR frequencies and vibrational assignments for the copolyamide.

Wavenumber [cm^−1^]	Assignment
3295	N–H stretch H-bonded
2923	Asymmetric CH_2_ stretch
2854	Symmetric CH_2_ stretch
1634	C=O stretch
1538	N–H bend
1463	CH_2_ scissors
1373	CH_2_ wagging
1201	CH_2_ twist-wagging
1168	CH_2_ wagging
685	N–H bend

**Table 4:** Surface energy data of the ternary system components.

Material	Surface Energy [mN/m]		
	$\gamma_{i}^{total}$	$\gamma_{i}^{d}$	$\gamma_{i}^{p}$
Fil-s	32.0	31.1	0.9
CoPA	39.5	32.7	6.7
Sepiolite PM15	33.5	30.5	3.0

**Table 5:** Calculated interfacial energies.

Material	Interfacial Energy [mN/m]	
	Harmonic Mean	Geometric Mean
Fil-s/CoPA	7.74	7.72
Fil-s/PM15	3.91	3.90
CoPA/PM15	1.59	0.87

**Table 6:** Thermal data relative to the cooling scan (where T_c,PE_, ΔH_c,PE_ and T_c,CoPA_, ΔH_c,CoPA_ are the crystallization temperatures and enthalpies of the PE and CoPA phases, respectively) for Fil-s, CoPA+20%PM15 masterbatch, and their blends.

SAMPLE	T_c,PE_ [°C]	ΔH_c,PE_ [J/g]	T_c,CoPA_ [°C]	ΔH_c,CoPA_[J/g]
Fil-s	112 ± 0.7	94.2 ± 3.5	–	–
CoPA+20%PM15	–	–	158 ± 1.8	36.4 ± 2.3
Fil-s+2.5%(CoPA+20%PM15)	112 ± 1.2	100.9 ± 2.3	–	–
Fil-s+5%(CoPA+20%PM15)	112 ± 0.9	97.5 ± 3.1	–	–

The crystallization enthalpies were normalized with respect to the total mass of the sample analyzed.

**Table 7:** Principal tensile mechanical properties of the ribbons made of the neat copolyamide, copolyamide masterbatch, Fil-s, and their blends.

SAMPLE	E [MPa]	εy [%]	σy [MPa]	εb [%]	σb [MPa]
* CoPA	540 ± 60	13.7 ± 2.1	39.9 ± 1.8	400 ± 30	38.8 ± 2.8
* CoPA + 20%PM15	1900 ± 80	18.4 ± 2.1	84.6 ± 3.2	36 ± 8	69.9 ± 3.2
Fil-s	440 ± 40	7.3 ± 0.2	15.8 ± 0.5	25 ± 4	13.0 ± 0.8
Fil-s + 2.5% CoPA	411 ± 7	14.0 ±0.6	15.3 ± 0.3	150 ± 50	11.8 ± 0.2
Fil-s + 2.5% (CoPA + 20%PM15)	474 ± 8	15.2 ± 0.5	15.8 ± 0.4	260 ± 90	12.4 ± 0.3
Fil-s + 5% CoPA	425 ± 5	12.8 ± 0.7	15.7 ± 0.4	90 ± 20	10.9 ± 0.5
Fil-s + 5% (CoPA + 20%PM15)	518 ± 8	12.9 ± 0.6	15.0 ± 0.5	50 ± 10	11.1 ± 0.6

* These specimens were produced at different processing conditions compared with the others reported in the table.
